# Efficient Perovskite Solar Cell with Improved Electron Extraction Based on SnO_2_/Phosphorene Heterojunction as Electron Transport Layer

**DOI:** 10.3390/ma18204771

**Published:** 2025-10-18

**Authors:** Min Li, Xin Yao, Jie Huang, Dawei Zhang

**Affiliations:** 1 College of Optical Electrical and Computer Engineering, University of Shanghai for Science and Technology, Shanghai 200093, China; limin3158@163.com; 2 Zhejiang Institute of Medical Device Supervision and Testing, Hangzhou 310018, China; 3 College of Optical and Electronic Technology, China Jiliang University, Hangzhou 310018, China; yaoxin_nk@126.com (X.Y.); huangjie@cjlu.edu.cn (J.H.)

**Keywords:** phosphorene, perovskite solar cells, electron transport layer, interface engineering, 2D materials

## Abstract

Due to its unique electrical and optical properties, as well as the tunable band structure based on thickness, 2D phosphorene recently emerged as a research hotspot and holds significant potential for applications across various fields. In this study, due to the special band structure and excellent electron transport performance of phosphorene, it formed a series structure with SnO_2_ as the electron transport layer of perovskite solar cells. Consequently, the photocurrent density was enhanced by approximately 20%, and the energy conversion efficiency was effectively elevated from 16.38% for pure SnO_2_ to 18.03% for the SnO_2_/phosphorene composite. Electrochemical measurements and spectral analyses revealed that the incorporation of phosphorene augmented electron mobility within the absorption layer, reduced the electron–hole recombination rate, and decreased the cell’s series resistance, thereby leading to improved efficiency of the perovskite solar cell. This research not only introduces a novel approach to enhancing solar cell efficiency but also paves a new pathway for the application of phosphorene.

## 1. Introduction

Metal halide perovskite has garnered significant attention as a highly promising light-absorbing material, owing to its advantageous band gap, exceptional absorption coefficient, and efficient charge carrier mobility [1]. Since the inception of perovskite solar cells, remarkable progress has been achieved, with energy conversion efficiencies surging from 3.8% to exceeding 27% [2,3,4]. This exponential improvement highlights the tremendous potential of perovskite solar cells. In particular, recent advances in semi-transparent perovskite solar cells have opened new pathways for tandem devices and building-integrated photovoltaics, placing additional importance on the optical and electrical design of functional layers such as the electron transport layer (ETL) [5]. However, despite their impressive performance, these cells face several challenges, including the thermal instability of the organic hole transport layer, the susceptibility of perovskite to environmental factors, and issues related to electronic extraction ability and electron mobility within the electron transport layer. These challenges collectively hinder the scalability and commercial viability of perovskite solar cells.

Among them, the electron transport layer (ETL) is crucial within the solar cell structure, as it efficiently conducts excited electrons from the absorption layer while suppressing hole recombination. Furthermore, it is essential to maintain a high optical transmittance [6,7,8,9,10,11,12]. In the beginning, mesoporous TiO_2_ has a high energy conversion efficiency due to its appropriate band gap and high light transmittance, making it an excellent material for electron transport layer. However, mesoporous TiO_2_ has a shielding effect on charge at the interface of perovskite, leading to a large amount of charge accumulation at the TiO_2_/perovskite interface, and its high sintering temperature [13] and photodegradation characteristics [14] limit its application and development. Zinc oxide (ZnO), with its exceptional electrical and optical properties, stands out as an ideal candidate for the electron transport layer. However, the surface characteristics of ZnO will degrade the perovskite layer, thereby impacting the efficiency of the perovskite solar cell [15,16,17]. To address this issue, Chen et al. introduced a layer of ZnS onto the ZnO surface, vulcanizing the ZnO/perovskite interface, forming a new electron transport pathway that expedites electron transport, reduces interfacial charge recombination, and enhances the cell stability, as validated through experimentation [18].

Recently, SnO_2_ has emerged as a prominent research material for the electron transport layer (ETL) in perovskite solar cells due to its exceptional properties. Compared with TiO_2_, SnO_2_ does not require high sintering temperature, allowing for the fabrication of SnO_2_ nanocrystal thin films through low-temperature solution processes. Moreover, SnO_2_ has higher electron mobility, more suitable band gap structure, and higher light transmittance than TiO_2_. And the environmental stability of SnO_2_ ensures the efficiency and stability of the cells [19,20,21]. While various two-dimensional materials including graphene and MXenes have been investigated for ETL applications, phosphorene stands out as a unique semiconductor with precisely positioned conduction and valence band edges that ideally align between those of SnO_2_ and the perovskite absorption layer. This strategic band alignment not only reduces the electron transport barrier at the SnO_2_/perovskite interface but also effectively blocks hole transfer to the ETL, thereby significantly suppressing carrier recombination at this critical interface. Therefore, the planar perovskite solar cell with SnO_2_ as the electron transport layer has the advantages of easier preparation and higher photoelectric conversion efficiency.

Based on the inherent advantages of SnO_2_, the performance of perovskite solar cells can be further improved by doping or compounding with other appropriate substances. Ye et al. [22] pioneered the use of low-temperature-treated zinc-doped SnO_2_ as the electron transport layer in perovskite solar cells, achieving an impressive energy conversion efficiency of 17.78%. Zinc doping is helpful for SnO_2_ to improve electrical conductivity, enhance electron extraction and transfer, inhibit charge recombination. Moreover, SnO_2_ doped with Zn has a wider exhaustion region, thus improving photovoltaic characteristics. Two-dimensional transition metal carbide has excellent electrical and optical properties.Yang et al. [13] used Ti_3_C_2_ and SnO_2_ composite as the electron transport layer of perovskite solar cells to improve the energy conversion efficiency. Ti_3_C_2_ provides an excellent charge transfer path, improves electron extraction and mobility, and reduces the electron transfer resistance at the ETL/perovskite interface, leading to increased photocurrent and higher Jsc and FF for the cells. Consequently, the photoelectric conversion efficiency of the cells is significantly enhanced.

In recent years, phosphorene has emerged as a prominent research focus in the field of two-dimensional (2D) materials. It can be separated from bulk black phosphorus by means of mechanical and liquid separation [23,24,25,26]. Phosphorene exhibits remarkable properties, including adjustable band gap (0.3–2.0 eV) according to thickness, high carrier mobility (>1000 cm^2^ V^−1^s^−1^) and anisotropy. These characteristics make phosphorene a promising candidate for application in photovoltaic devices [27,28]. Here, we apply phosphorene and SnO_2_ as electron transport layer to perovskite solar cells. The structure of the solar cell is: FTO (Fluorine-doped Tin Oxide Glass)/SnO_2_/Phosphorene/Perovskite/Spiro-OMeTAD/Au. Phosphorene is between the perovskite absorption layer and SnO_2_. Since the conduction band position of phosphorene is lower than that of perovskite and higher than that of SnO_2_, it acts as a buffer to improve the efficiency of electron transfer and avoid the high charge recombination rate caused by too large band order. Meanwhile, the valence band position of phosphorene is lower than that of perovskite, which plays a role in preventing the transfer of holes and improving the separation efficiency of electrons and holes. In addition, the high carrier mobility of phosphorene also contributes to improving the energy conversion efficiency of the cells. We have conducted tests and confirmed that the addition of phosphorene enhances the energy conversion efficiency of perovskite solar cells from 16.38% to 18.03%, demonstrating a significant effect. This study provides new ideas for the application of phosphorene and enhancement of the solar cell efficiency.

## 2. Materials and Methods

### 2.1. Preparation of Phosphorene

To utilize phosphorene, it is essential to exfoliate few-layer or even single-layer phosphorene from black phosphorus [29]. We purchased 1 g of lumpy black phosphorus (Zhongke Technology Experimental Materials Company, Beijing, China), a specific amount was taken from a N2 atmosphere glove box (Mikolana Industrial Intelligent Technology Co., Ltd., Shanghai, China) and placed into a mortar (Lichen Technology Co., Ltd., Changsha, China) for grinding. After being ground into a powder, 25 mg was weighed out into a 150 mL beaker (Hangzhou Jiachen Chemical Co., Ltd., Hangzhou, China), and 100 mL of NMP solution was added to mix. Then, tightly wrap the beaker with tin foil and remove it from the glove box. Subsequently, the black phosphorus/NMP mixture was subjected to ultrasonic treatment using a powerful ultrasonic machine (Ningbo Xinzhi Biotechnology Co., Ltd., Ningbo, China) in the laboratory. The liquid was directly probed by inserting the oscillator (Jintan District Xicheng Xinrui Instrument Factory, Changzhou, China), while the beaker containing it was positioned within a cooling water circulation system (Putec Refrigeration Equipment Technology Co., Ltd., Shanghai, China) to enhance phosphorene yield. Ultrasonic conditions: To prevent rapid temperature rise from affecting exfoliation effect of phosphorene, intermittent ultrasonic treatment was applied for 12 h (ultrasonic for 4 s/stop for 4 s). The powerful ultrasonic machine used had an output power of 972 W. The cooling water temperature was maintained at 0 °C. The ultrasound process was conducted in darkness.

### 2.2. Preparation of SnO_2_/Phosphorene Compound

To assess the enhancement in photoelectrochemistry properties of SnO_2_ upon the incorporation of phosphorene, it is necessary to fabricate the SnO_2_/phosphorene composite and subsequently conduct its characterization. The phosphorene was initially prepared by dispersing bulk black phosphorus powder in N-methyl-2-pyrrolidone (NMP) (Shanghai McLean Biochemical Technology Co., Ltd., Shanghai, China) at a concentration of 0.2 mg/mL, followed by probe sonication at 1080 W total power in pulsed mode (4 s on/4 s off) for 12 h under ice-bath conditions.

First of all, we centrifugally treated the ultrasonic phosphorene/NMP suspension to obtain the phosphorene nanosheet with appropriate size and thickness: the suspension was centrifuged at 6000 r/min for 15 min to remove thicker particles, and the resulting supernatant was then centrifuged again at 10,000 r/min for another 15 min, settling phosphorene nanosheets with a thickness of approximately 2 to 3 nm. Removing the supernatant and taking the precipitation, its proceeds to a certain proportion to join SnO_2_ hydrocolloidal dispersion (Shanghai Jizhi Biochemical Technology Co., Ltd., Shanghai, China) (15% in H_2_O colloidal dispersion). The mixture is then subjected to ultrasonic treatment in an ice bath for 30 min using an ultrasonic cleaner (Shenzhen Jiemeng Cleaning Equipment Co., Ltd., Shenzhen, China). This process ultimately yields the SnO_2_/phosphorene compound.

### 2.3. Preparation of Perovskite Solar Cells Containing Phosphorene

Because the perovskite will be dissolved by NMP, we need to wash the separated phosphorene/NMP dispersion solution with toluene when the phosphorene is rotated to the cell as an electron transport layer. First, we added 5 mL toluene (Shanghai Lingfeng Chemical Reagent Co., Ltd., Shanghai, China) to the phosphorene/NMP dispersion in a test tube, then centrifuged at 10,000 r/min for 15 min. We removed the supernatant until only 1 mL remained, and then employed ultrasound for 15 mins using an ultrasonic cleaner. We repeated this process 3 times, and the obtained phosphorene/toluene dispersion solution was used for spin-coating preparation of perovskite solar cell. The cell structure was FTO/SnO_2_/Phosphorene/Perovskite/Spiro-OMeTAD/Au.

### 2.4. Material Characterization

The thickness and lateral dimensions of the exfoliated phosphorene were determined by atomic force microscopy (AFM) (Veeco Precision Instrument Co., Ltd., New York, NY, USA) The morphology and successful formation of the SnO_2_/phosphorene composite were confirmed using transmission electron microscopy (TEM). (Japan Electronics Corporation, Tokyo, Japan). X-ray photoelectron spectroscopy (XPS) was employed to examine the chemical states and evaluate the stability against oxidation after exfoliation and composite formation. Raman spectroscopy (HORIBA Corporation, Tokyo, Japan) was applied to monitor the structural integrity and possible oxidation of phosphorene. The charge separation behavior and carrier lifetime were investigated by photoluminescence (PL) and time-resolved photoluminescence (TRPL) measurements. Finally, the enhanced photoelectrochemical performance induced by phosphorene incorporation was systematically evaluated using electrochemical impedance spectroscopy (EIS), incident photon-to-electron conversion efficiency (IPCE), Mott-Schottky analysis, UV-Vis spectroscopy, and photocurrent (I-T) measurements.

## 3. Results and Discussion

**Figure 1:**
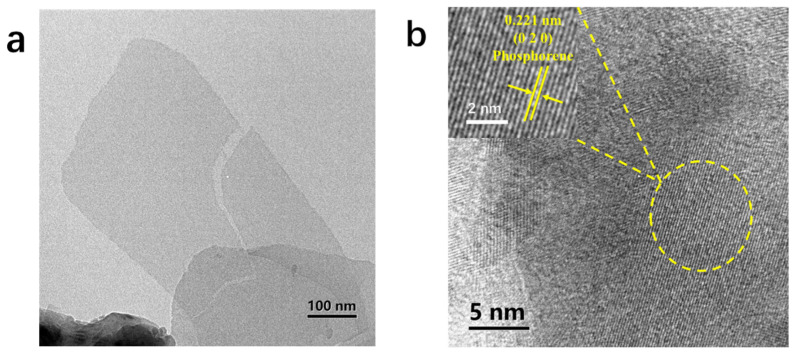

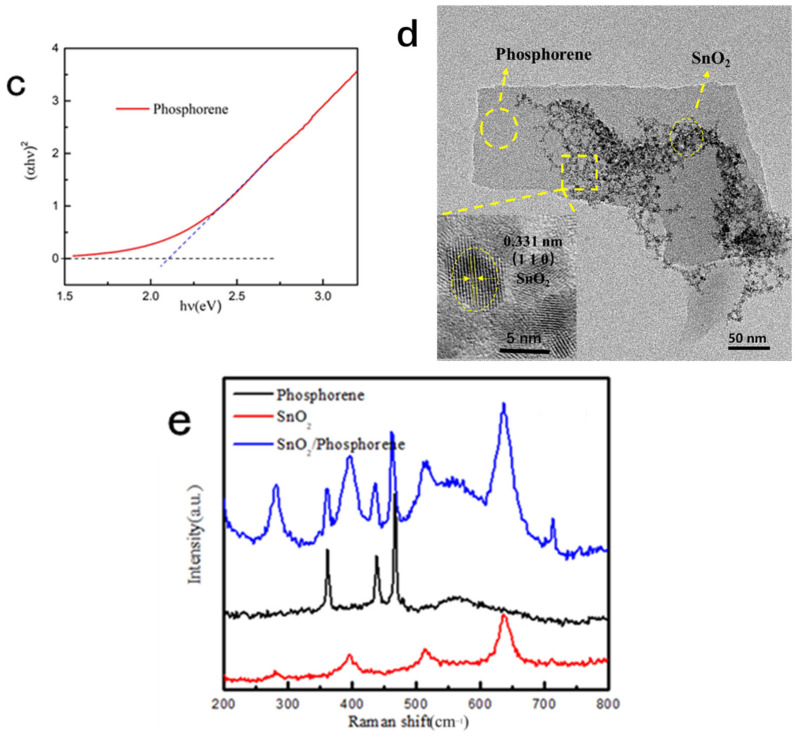
Morphological and structural characterization of phosphorene and the SnO_2_/phosphorene composite. (**a**) TEM image of exfoliated phosphorene nanosheets. (**b**) HRTEM image of a phosphorene nanosheet. (**c**) Tauc plot derived from UV-Vis spectroscopy indicating the optical bandgap of phosphorene. (**d**) TEM image of the SnO_2_/phosphorene hybrid material. (**e**) Raman spectra of pristine phosphorene, SnO_2_, and the SnO_2_/phosphorene composite.

TEM and HRTEM were used to investigate the microstructure of the phosphorene and the composition of the SnO_2_/phosphorene composite. Figure 1a shows the TEM after the successful extraction of phosphorene, we can see the sheet structure is clear compared with the unsuccessfully exfoliated black phosphorus in the lower left corner, indicating the thinness of the phosphorene. The uniform color across the entire image suggests consistent thickness, with a lateral dimension exceeding 100 nm, demonstrating remarkable exfoliation effectiveness. Figure 1b presents the HRTEM result of this region. The image reveals the neat lattice fringe, which, upon measurement and comparison with literature, are identified as the (0 2 0) [30] crystal surface of P. The position of the Raman peak observed in the phosphorene’s Raman spectrum (depicted in Figure 1e) aligns closely with the values reported in the previous literature [9], thereby providing solid evidence that the material in question is indeed phosphorene successfully obtained through exfoliation processes. Then, the band gap of phosphorene was characterized by UV-vis, and it was concluded from Figure 1c that the band gap of phosphorene was about 2.1 eV, which met our experimental requirements. Figure 1d is the TEM of SnO_2_/phosphorene. It can be seen that the nanosheet with light color and the thin flake at the bottom are phosphorene nanosheet, and the black particles are SnO_2_. We also identified the (1 1 0) crystal plane of SnO_2_ from HRTEM. However, the lattice fringes of phosphorene were not clearly observed, which can likely be attributed to its ultra-thin layered structure and comparatively low crystallinity following exfoliation. This phenomenon is consistent with previously reported challenges in resolving lattice structures of few-layer phosphorene via conventional TEM. But according to the TEM image of the phosphorene shown in Figure 1a, we can infer that the sheet structure at the bottom of the figure is phosphorene. From the image, it is evident that phosphorene and SnO_2_ have successfully combined following the experiment. Figure 1e presents the Raman spectroscopy analyses of phosphorene, SnO_2_ and their composite phases. The data reveals that the Raman peak positions of both phosphorene and SnO_2_ align with those of the SnO_2_/phosphorene composite, confirming the successful integration of the two materials. It is also stated in the pertinent literature that when the peak intensity ratio of the phosphorene at 361 and 462 exceeds 0.6, one can be ascertain that phosphorene remains oxidized [31]. The respective intensities of the two sections of the SnO_2_/phosphorene were measured as 1588 and 2311, yielding a calculated ratio of 0.67 (>0.6), thereby confirming that the phosphorene within the composite was free from oxidation.

In summary, the exfoliation method and extraction conditions were progressively optimized through adjustments to ultrasonic intensity, exfoliation liquid concentration, centrifugal speed, among other factors. Furthermore, an array of material characterization techniques was used to analyze both the phosphorene and SnO_2_/phosphorene compound. The results indicated that the fabricated phosphorene could achieve a thickness of 4–6 layers. Upon mixing with SnO_2_ hydrocolloidal dispersion, SnO_2_ particles were effectively dispersed on the surface, ensuring the stability of the easily oxidized phosphorene. of the phosphorene and the easily oxidized phosphorene could be kept stable. These findings facilitate the further application of phosphorene in the electron transport layer of perovskite solar cells.

**Figure 2:**
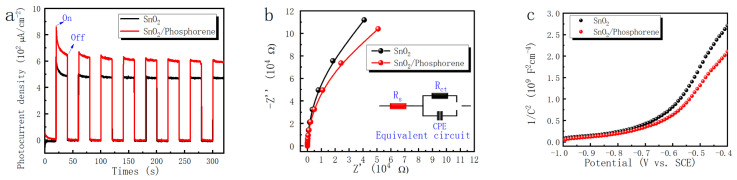
Photoelectrochemical and electronic characterization of SnO_2_ and SnO_2_/phosphorene composite. (**a**) Transient photocurrent (I-T) curves of SnO_2_ and SnO_2_/phosphorene under simulated sunlight illumination. (**b**) Electrochemical impedance spectra presented as Nyquist plots. (**c**) Mott-Schottky plots measured at a specific frequency.

To investigate the enhancement of photoelectrochemical properties in SnO_2_ upon incorporation of phosphorene, a comprehensive series of photoelectrochemical tests were conducted. Although SnO_2_ inherently exhibits relatively superior electrochemical performance [32], the incorporation of phosphorene resulted in measurable and consistent improvements across multiple performance metrics. Figure 2 illustrates the electrical performance assessment of SnO_2_/phosphorene electron transport layers, as evaluated by electrochemical impedance spectroscopy under dark conditions at room temperature with a 0 V DC bias, 10 mV AC amplitude, and a frequency range of 100 kHz to 0.1 Hz. As shown in Figure 2a, the photocurrent density of SnO_2_ increased by approximately 20% after the incorporation of phosphorene, indicating significantly enhanced photoresponse. Given that photocurrent density is influenced by charge transfer efficiency and carrier concentration, electrochemical impedance spectroscopy (EIS) and Mott-Schottky analyses were also conducted. The EIS results (Figure 2b) show that the composite exhibited a smaller arc radius, suggesting improved charge transfer and reduced recombination resistance. The Mott-Schottky plot (Figure 2c) confirms n-type semiconductor behavior for both materials, and the decreased slope for the SnO_2_/phosphorene sample indicates an increase in charge carrier density [33], consistent with the enhanced photoelectrochemical performance.

**Figure 3:**
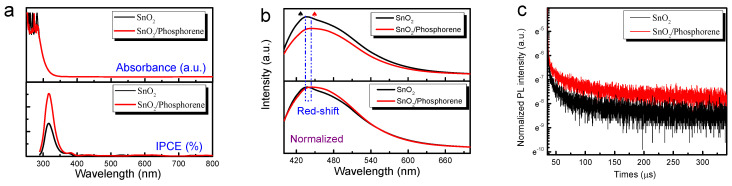
Optical and spectroscopic characterization of SnO_2_ and SnO_2_/phosphorene composite. (**a**) UV-Vis absorption spectra and incident photon-to-current efficiency (IPCE) curves. (**b**) Steady-state photoluminescence (PL) spectra. (**c**) Time-resolved photoluminescence (TRPL) decay curves.

Figure 3 presents the optical performance test results of SnO_2_/phosphorene. As shown in Figure 3a, the phosphorene has a negligible impact on the light absorption of SnO_2_, likely due to the very small amount of phosphorene added. The IPCE directly reflects the separation degree of electrons and holes as well as the photoelectric conversion capability after the material is exposed to light. As depicted in Figure 3a, the inclusion of phosphorene into SnO_2_ markedly enhances the IPCE value, indicating that phosphorene facilitates the separation of photogenerated carriers. PL is associated with the charge transfer performance of the photoinduced electrons and holes, enabling the investigation of excited-state interactions between SnO_2_ and phosphorene. Figure 3b illustrates the intrinsic peak of SnO_2_ at a wavelength of 434 nm. SnO_2_/phosphorene exhibit lower fluorescence emission due to the formation of complex substances [34]. The spectrum shows a slight red shift originating from the interaction between SnO_2_ and phosphorene, compared with pure SnO_2_. The significantly longer carrier lifetime in the SnO_2_/Phosphorene sample, as evidenced by the slower PL decay kinetics in Figure 3c, clearly demonstrates more effective charge carrier separation at the perovskite/ETL interface.

**Figure 4:**
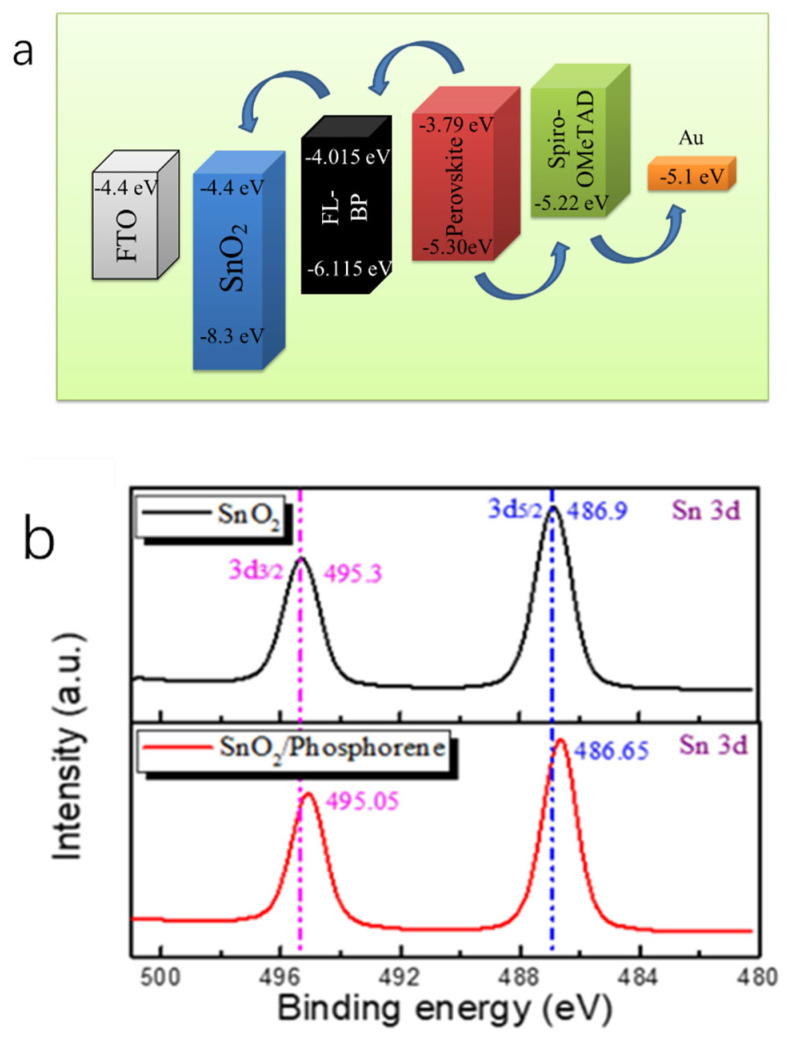
(**a**). Energy level positions of various parts of perovskite solar cells [9,27]; (**b**). XPS spectral analysis of Sn element.

Based on the enhanced charge transport properties of the SnO_2_/phosphorene composite, we fabricated n-i-p structured perovskite solar cells (PSCs) with FTO/SnO_2_/phosphorene as the electron transport layer (ETL), perovskite as the light absorber, Spiro-OMeTAD as the hole transport layer (HTL), and Au as the electrode. The energy level alignment of each functional layer, derived from experimental measurements and literature references [9,27], is illustrated in Figure 4b. The conduction band (CB) and valence band (VB) of the perovskite absorber are located at approximately −3.79 eV and −5.30 eV. These are higher than the energy levels of phosphorene (CB: −4.4 eV; VB: −6.115 eV) and SnO_2_ (CB: −4.4 eV; VB: ≈ −8.3 eV), facilitating efficient electron extraction from perovskite into the ETL while effectively blocking hole transfer. Meanwhile, the valence band of perovskite (−5.30 eV) is slightly lower than that of Spiro-OMeTAD (−5.22 eV), which promotes hole transport to the HTL and suppresses electron leakage. The intermediate energy levels of phosphorene play a critical role in bridging perovskite and SnO_2_, serving as a buffer that reduces the electron injection barrier and suppresses interfacial recombination, thereby significantly improving charge extraction and overall device performance.

Here, we employed XPS (Figure 4b) to investigate whether electrons can be transferred to SnO_2_ through phosphorene. The observed shift in the Sn element peak towards lower binding energy upon the introduction of phosphorene suggests an increase in the electron cloud density around Sn, thereby indicating electron transfer from phosphorene to SnO_2_. Therefore, the phosphorene fulfilled its intended role effectively, facilitating electron–hole pair separation and transport.

**Figure 5:**
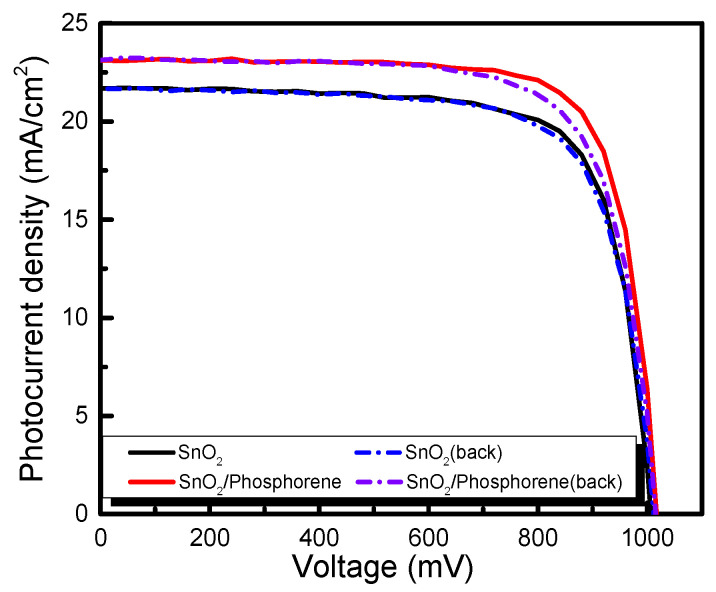
J-V curves of perovskite solar cells with SnO_2_ and SnO_2_/phosphorene as electron transport layer.

To investigate the enhancement effect of phosphorene on cell performance, a comparative analysis was conducted between cells with and without phosphorene doping. The measured J-V curve was presented in Figure 5, and a comparison of the derived parameters is summarized in Table 1. The curve clearly illustrates a significant increase in the short-circuit photocurrent density following the incorporation of phosphorene, with an enhancement of approximately 1.37 mA/cm^2^. And as shown in Table 1, the series resistance of the perovskite solar cell was marginally decreased, leading to an enhancement in energy conversion efficiency to 18.03%. To sum up, while SnO_2_ serves as a good conductive base, the distinctive energy level alignment and efficient electron transfer capability of phosphorene enable the SnO_2_/phosphorene composite to function as a superior electron transport layer, ultimately enhancing the energy conversion efficiency of perovskite solar cells. This demonstrates the promising potential of phosphorene as a novel two-dimensional semiconductor in photoelectrochemical applications.

## 4. Conclusions

Phosphorene has emerged as a focal point in the research of two-dimensional materials, owing to its exceptional electron transport properties and unique energy level structure. This paper presents an effective method for enhancing the performance of perovskite solar cells by employing a composite of 2D phosphorene and SnO_2_ as the electron transport layer. Since the conduction band and valence band levels of phosphorene lie between those of SnO_2_ and perovskite, it not only facilitates more efficient transfer of photo-induced electrons to the electron transport layer but also effectively inhibits hole influx, thereby preventing the accumulation of electrons at the interface between the electron transport layer and the perovskite. Jsc is increased and Rs is decreased, leading to an improvement in the energy conversion efficiency of the cell. Therefore, to enhance the energy conversion efficiency of perovskite solar cells, we can select materials with an appropriate energy level structure and high carrier mobility for the electron transport layer, thereby achieving more efficient charge transfer. While recent studies on SnO_2_ ETL modification using doping strategies or other 2D materials like MXenes have reported higher absolute PCE values, our phosphorene-based approach offers distinct advantages of solution processability and optimal band alignment.

Future studies should systematically investigate the operational stability of phosphorene-based devices under illumination, thermal stress, and ambient conditions. The scalability of phosphorene synthesis and processing also requires further evaluation. Moreover, the tunable optoelectronic properties of phosphorene offer promising opportunities for application in semi-transparent and tandem perovskite solar cells.

## Figures and Tables

**Table 1:** Comparison of performance parameters of solar cells.

	Jsc (mA/cm^2^)	Voc (mV)	FF	The Eff (%)	Rsh (Ω·cm^2^)	Rs (Ω·cm^2^)
phosphorene/SnO_2-_fore	23.14	1020.13	0.73	17.28	2982.59	2.80
phosphorene/SnO_2-_back	23.10	1020.13	0.77	18.03	2467.55	2.47
SnO_2_-fore	21.72	1020.14	0.73	16.07	1545.97	3.12
SnO_2_-back	21.73	1020.14	0.74	16.38	1340.80	2.70

## Data Availability

The original contributions presented in this study are included in the article. Further inquiries can be directed to the corresponding author.

## References

[1] Liao Y.-H., Chang Y.-H., Lin T.-H., Lee K.-M., Wu M.-C. (2024). Recent Advances in Metal Oxide Electron Transport Layers for Enhancing the Performance of Perovskite Solar Cells. Materials.

[2] National Renewable Energy Laboratory (NREL) Research Cell Efficiency Records. https://www.nrel.gov/pv/cell-efficiency.

[3] Sahli F., Werner J., Kamino B.A., Bräuninger M., Monnard R., Paviet-Salomon B., Barraud L., Ding L., Leon J.J.D., Sacchetto D. (2018). Fully textured monolithic perovskite/silicon tandem solar cells with 25.2% power conversion efficiency. Nat. Mater..

[4] Xu Z., Lan Z., Chen F., Yin C., Wang L., Li Z., Yan L., Ji J. (2024). Regulating TiO_2_ Deposition Using a Single-Anchored Ligand for High-Efficiency Perovskite Solar Cells. Materials.

[5] Spampinato C., Calogero G., Mannino G., Valastro S., Smecca E., Arena V., La Magna P., Bongiorno C., Fazio E., Alberti A. (2025). A Sputtered Gig-Lox TiO_2_ Sponge Integrated with CsPbI3:EuI2 for Semitransparent Perovskite Solar Cells. J. Phys. Chem. C.

[6] Damgaci E., Kartal E., Gucluer F., Seyhan A., Kaplan Y. (2024). Impact of Temperature Optimization of ITO Thin Film on Tandem Solar Cell Efficiency. Materials.

[7] Singh R., Giri A., Pal M., Thiyagarajan K., Kwak J., Lee J.-J., Jeong U., Cho K. (2019). Perovskite solar cells with an MoS_2_ electron transport layer. J. Mater. Chem. A.

[8] Tavakoli M.M., Tavakoli R., Yadav P., Kong J. (2019). A graphene/ZnO electron transfer layer together with perovskite passivation enables highly efficient and stable perovskite solar cells. J. Mater. Chem. A.

[9] Roose B., Johansen C.M., Dupraz K., Jaouen T., Aebi P., Steiner U., Abate A. (2018). A Ga-doped SnO_2_ mesoporous contact for UV stable highly efficient perovskite solar cells. J. Mater. Chem. A.

[10] Xu S., Yang L., Wang Z., Li F., Zhang X., Zhou J., Lv D., Ding Y., Sun W. (2025). Few-Layered Black Phosphorene as Hole Transport Layer for Novel All-Inorganic Perovskite Solar Cells. Materials.

[11] Zhou Y., Yang S., Yin X., Han J., Tai M., Zhao X., Chen H., Gu Y., Wang N., Lin H. (2019). Enhancing electron transport via graphene quantum dot/ SnO_2_ composites for efficient and durable flexible perovskite photovoltaics. J. Mater. Chem. A.

[12] Wang S., Zhu Y., Liu B., Wang C., Ma R. (2019). Introduction of carbon nanodots into SnO_2_ electron transport layer for efficient and UV stable planar perovskite solar cells. J. Mater. Chem. A.

[13] Yang L., Dall’Agnese Y., Hantanasirisakul K., Shuck C.E., Maleski K., Alhabeb M., Chen G., Gao Y., Sanehira Y., Jena A.K. (2019). SnO_2_-Ti_3_C_2_ MXene electron transport layers for perovskite solar cells. J. Mater. Chem. A.

[14] Lee M.M., Teuscher J., Miyasaka T., Murakami T.N., Snaith H.J. (2012). Efficient Hybrid Solar Cells Based on Meso-Superstructured Organometal Halide Perovskites. Science.

[15] Shin S.S., Yeom E.J., Yang W.S., Hur S., Kim M.G., Im J., Seo J., Noh J.H., Seok S.I. (2017). Colloidally prepared La-doped BaSnO_3_ electrodes for efficient, photostable perovskite solar cells. Science.

[16] Li S., Zhang P., Wang Y., Sarvari H., Liu D., Wu J., Yang Y., Wang Z., Chen Z.D. (2017). Interface engineering of high efficiency perovskite solar cells based on ZnO nanorods using atomic layer deposition. Nano Res..

[17] Yang J.L., Siempelkamp B.D., Mosconi E., De Angelis F., Kelly T.L. (2015). Origin of the Thermal Instability in CH_3_NH_3_PbI_3_ Thin Films Deposited on ZnO. Chem. Mat..

[18] Chen R.H., Cao J., Duan Y., Hui Y., Chuong T.T., Ou D.H., Han F.M., Cheng F.W., Huang X.F., Wu B.H. (2019). High-Efficiency, Hysteresis-Less, UV-Stable Perovskite Solar Cells with Cascade ZnO-ZnS Electron Transport Layer. J. Am. Chem. Soc..

[19] Totolhua E.P., López J.C., Lara A.B., Leyva K.M., Reyes A.C.P., Flores-Méndez J., López J.A.L. (2023). Numerical Simulation of an Inverted Perovskite Solar Cell Using a SiO_x_ Layer as Down-Conversion Energy Material to Improve Efficiency and Stability. Materials.

[20] Song J., Zheng E., Bian J., Wang X.-F., Tian W., Sanehira Y., Miyasaka T. (2015). Low-temperature SnO_2_-based electron selective contact for efficient and stable perovskite solar cells. J. Mater. Chem. A.

[21] Hendra W.M., Nagaya N., Hibi Y., Yoshida N., Sugiura T., Vafaei S., Manseki K. (2024). Facile Synthesis, Sintering, and Optical Properties of Single-Nanometer-Scale SnO_2_ Particles with a Pyrrolidone Derivative for Photovoltaic Applications. Materials.

[22] Ye H., Liu Z., Liu X., Sun B., Tan X., Tu Y., Shi T., Tang Z., Liao G. (2019). 17.78% efficient low-temperature carbon-based planar perovskite solar cells using Zn-doped SnO_2_ electron transport layer. Appl. Surf. Sci..

[23] Guo Z., Zhang H., Lu S., Wang Z., Tang S., Shao J., Sun Z., Xie H., Wang H., Yu X. (2015). From Black Phosphorus to Phosphorene: Basic Solvent Exfoliation, Evolution of Raman Scattering, and Applications to Ultrafast Photonics. Adv. Funct. Mater..

[24] Yasaei P., Kumar B., Foroozan T., Wang C.H., Asadi M., Tuschel D., Indacochea J.E., Klie R.F., Salehi-Khojin A. (2015). High-Quality Black Phosphorus Atomic Layers by Liquid-Phase Exfoliation. Adv. Mater..

[25] Pei J., Gai X., Yang J., Wang X., Yu Z., Choi D.-Y., Luther-Davies B., Lu Y. (2016). Producing air-stable monolayers of phosphorene and their defect engineering. Nat. Commun..

[26] Xia F., Wang H., Jia Y. (2014). Rediscovering black phosphorus as an anisotropic layered material for optoelectronics and electronics. Nat. Commun..

[27] Batmunkh M., Bat-Erdene M., Shapter J.G. (2016). Phosphorene and Phosphorene-Based Materials—Prospects for Future Applications. Adv. Mater..

[28] Lee T.H., Kim S.Y., Jang H.W. (2016). Black Phosphorus: Critical Review and Potential for Water Splitting Photocatalyst. Nanomaterials.

[29] He R., Hua J., Zhang A., Wang C., Peng J., Chen W., Zeng J. (2017). Molybdenum Disulfide-Black Phosphorus Hybrid Nanosheets as a Superior Catalyst for Electrochemical Hydrogen Evolution. Nano Lett..

[30] Zhu M., Osakada Y., Kim S., Fujitsuka M., Majima T. (2017). Black phosphorus: A promising two dimensional visible and near-infrared-activated photocatalyst for hydrogen evolution. Appl. Catal. B Environ..

[31] Hanlon D., Backes C., Doherty E., Cucinotta C.S., Berner N.C., Boland C., Lee K., Harvey A., Lynch P., Gholamvand Z. (2015). Liquid exfoliation of solvent-stabilized few-layer black phosphorus for applications beyond electronics. Nat. Commun..

[32] Chen C., Jiang Y., Guo J.L., Wu X.Y., Zhang W.H., Wu S.J., Gao X.S., Hu X.W., Wang Q.M., Zhou G.F. (2019). Solvent-Assisted Low-Temperature Crystallization of SnO_2_ Electron-Transfer Layer for High-Efficiency Planar Perovskite Solar Cells. Adv. Funct. Mater..

[33] Wang G., Wang H., Ling Y., Tang Y., Yang X., Fitzmorris R.C., Wang C., Zhang J.Z., Li Y. (2011). Hydrogen-treated TiO_2_ nanowire arrays for photoelectrochemical water splitting. Nano Lett..

[34] Shi H., Ge S., Wang Y., Gao C., Yu J. (2019). Wide-Spectrum-Responsive Paper-Supported Photoelectrochemical Sensing Platform Based on Black Phosphorus-Sensitized TiO_2_. ACS Appl. Mater. Interfaces.

